# Disciplinary trends in spatial concept associations: a multifaceted analysis of student responses

**DOI:** 10.3389/fpsyg.2026.1745600

**Published:** 2026-05-20

**Authors:** Ali Özkaya, Saim Turan, Adem Uzun, Osman Akhan

**Affiliations:** 1 Department of Mathematics and Science Education, Faculty of Education, Akdeniz University, Antalya, Türkiye; 2 Department of Social Studies Education, Faculty of Education, Akdeniz University, Antalya, Türkiye; 3 Department of Gifted Education, Faculty of Education, Cumhuriyet University, Sivas, Türkiye

**Keywords:** associative methods, disciplinary orientation, geography, mathematics, spatial assessment, spatial concept associations

## Abstract

**Introduction:**

This study addresses a critical need to better understand how students conceptualize spatial knowledge across disciplinary contexts, a key issue for improving spatial thinking in education. Although spatial cognition has been widely studied, existing research predominantly relies on standardized performance-based assessments, which provide limited insight into how learners cognitively organize and associate spatial concepts across disciplines. This reveals a significant gap in capturing the conceptual and interdisciplinary dimensions of spatial understanding.

**Methods:**

To address this limitation, a stimulus-based word association task was administered to 417 middle school students in Türkiye, yielding 16,680 responses categorized into four disciplinary orientations: mathematics, geography, both, or neither. Reliability was established through expert coding and Fleiss’ Kappa, and analyses included chi-square tests, principal component analysis, hierarchical clustering, and non-parametric comparisons.

**Results:**

The findings reveal systematic disciplinary patterns in students’ conceptualizations: while slope, scale, and area are predominantly associated with mathematics, concepts such as coordinate, location, and graph function as interdisciplinary connectors. In contrast, environment and distance show weak disciplinary anchoring, suggesting more context-dependent interpretations. Significant differences were also observed across gender, transportation mode, and disciplinary orientation.

**Discussion:**

By introducing an associative-response approach, this study extends current spatial cognition research beyond performance-based measures and provides empirical evidence for discipline-oriented patterns in spatial thinking. The findings suggest that spatial cognition is not solely a domain-general ability but is shaped by educational experiences and disciplinary exposure, offering a refined conceptual perspective for understanding spatial intelligence in educational contexts.

## 1 Introduction

Spatial skills are essential for how individuals perceive, interpret, and make decisions about their environments, with wide-ranging implications for both education and society. They support cognitive processes such as wayfinding, locating objects, estimating distances, and interpreting spatial relationships (Ishikawa, 2021; Vasilyeva and Lourenco, 2012; Bellmund et al., 2018). Beyond physical navigation, spatial cognition also encompasses abstract reasoning, supporting learning across multiple domains. In educational contexts, research often frames these abilities within the construct of spatial intelligence, a multidimensional competency involving mental organization, transformation, and problem-solving with spatial information. Core abilities such as mental rotation, visualization, and navigation are not only vital for everyday functioning but also for advanced reasoning in mathematics, science, and geography (Malanchini et al., 2020; Ishikawa, 2021; Hsing et al., 2022).

Spatial skills exhibit unitary cognitive architecture and high heritability, reinforcing their role as a distinct cognitive domain (Malanchini et al., 2020; Geary, 2022). Within Gardner’s theory of multiple intelligences, spatial intelligence is recognized as a separate domain that underpins disciplinary practices, particularly in mathematics and geography, where spatial representations and relationships are central to conceptual understanding (Mix, 2019; Hsing et al., 2022). Moreover, research has shown that spatial skills predict STEM success, with their development shaped by both biological maturation and educational exposure to symbolic representations such as maps, graphs, and spatial models (Vasilyeva and Lourenco, 2012; Yu et al., 2022).

Developing spatial skills in education requires more than technical mastery of maps or graphs; it also involves building the conceptual capacity to connect spatial information across disciplinary contexts and interpret spatial terminology appropriately. Yet empirical evidence remains limited on how students associate spatial concepts with academic disciplines. A notable methodological gap exists in assessing how learners classify concepts such as coordinate, area, graph, and location with mathematics or geography (Chen et al., 2020). Because these concepts are inherently interdisciplinary, understanding their categorization offers insight into students’ conceptual frameworks and disciplinary orientations.

Despite the growing body of research on spatial cognition, several critical limitations remain. First, existing studies provide inconsistent evidence regarding the disciplinary nature of spatial thinking. While some research conceptualizes spatial ability as a domain-general cognitive skill, other studies emphasize its domain-specific manifestations across fields such as mathematics, geography, and science, suggesting the coexistence of both general and discipline-bound spatial processes (Chen et al., 2020; Atit et al., 2020; Gilligan-Lee et al., 2022). Second, empirical findings on factors influencing spatial cognition, particularly gender differences and contextual variables, remain inconclusive and highly dependent on task characteristics, measurement instruments, and learning environments (Lauer et al., 2019; Nazareth et al., 2019; Bartlett and Camba, 2023). Third, most studies rely heavily on standardized psychometric tests, most notably mental rotation tasks, which tend to overrepresent narrow aspects of spatial ability and fail to capture how learners organize, interpret, and apply spatial concepts within authentic disciplinary contexts (Frick and Pichelmann, 2023; Ishikawa and Newcombe, 2021; Harris, 2023). Consequently, a significant gap persists in understanding the conceptual and associative dimensions of spatial thinking, particularly in educational settings where spatial knowledge is distributed across multiple subject areas.

To address these gaps, recent studies advocate innovative approaches for examining how individuals conceptualize spatial ideas across domains. One promising method analyzes participants’ associative responses to spatial concepts, enabling researchers to detect patterns of disciplinary orientation and explore how these vary by demographic and educational variables (Mix, 2019). This approach also responds to broader concerns about existing spatial assessments, which are often inaccessible, inconsistently defined, and lack robust psychometric validation (Uttal et al., 2024). Furthermore, evidence from psychometric modeling demonstrates that specific item characteristics significantly shape task difficulty, underscoring the need for diversified and well-validated methods of assessing spatial reasoning (Shi et al., 2023).

In response to these limitations, the present study adopts an alternative conceptual and methodological approach by examining spatial cognition through associative responses rather than performance-based tasks. By focusing on how learners categorize spatial concepts across disciplinary domains, this approach moves beyond traditional psychometric assessments and provides insight into the underlying cognitive orientations that shape spatial understanding. In doing so, it contributes to ongoing theoretical discussions by providing empirical evidence that spatial cognition may reflect discipline-oriented patterns shaped by educational experience, rather than a purely domain-general structure. Building on this perspective, the study investigates the disciplinary tendencies underlying students’ spatial concept associations. Using stimulus-based associative tasks, expert-coded classifications, and statistical and multivariate analyses, including Fleiss’ Kappa, residual analysis, PCA, clustering, and non-parametric comparisons, the study examines how middle school students in Türkiye associate spatial concepts with mathematics, geography, both, or neither. In doing so, it aims to reveal the cognitive structures guiding spatial conceptualization across domains and contribute a novel measurement approach to research on spatial cognition and interdisciplinary learning.

### 1.1 Theoretical framework

A theoretical framework provides the interpretive lens that shapes the inquiry and ensures coherence throughout the study (Cai et al., 2020; Kivunja, 2018). In this research, the investigation of students’ spatial concept associations is grounded in three complementary perspectives: theories of multiple intelligences, research on spatial cognition, and approaches to interdisciplinary thinking. Together, these frameworks support the rationale for examining how learners conceptualize and classify spatial concepts within mathematics, geography, both, or neither.

### 1.2 Theories underpinning spatial concept associations

This study draws principally on Gardner’s (1983) theory of multiple intelligences, in which spatial intelligence is recognized as a distinct cognitive domain. According to Gardner, spatial intelligence involves the capacity to mentally manipulate, transform, and represent spatial configurations, abilities that are critical for tasks such as orientation, visualization, and navigation. These capabilities are foundational not only for understanding spatial phenomena but also for interpreting academic concepts across disciplines, including mathematical constructs such as geometry, graphing, and coordinate systems, as well as geographic constructs like map reading, spatial scale, and location (Mix, 2019; Gardner, 1983; Yu et al., 2022). Gardner’s framework therefore provides a conceptual basis for investigating how individuals cognitively affiliate spatial concepts with disciplines.

Support for this theoretical orientation is also found in psychological and cognitive science research, which underscores the distinctiveness of spatial cognition from general intelligence. Empirical evidence shows that spatial skills exhibit a unitary yet independent cognitive structure, with strong heritability and predictive power for academic success in STEM domains (Malanchini et al., 2020; Geary, 2022). Within this perspective, examining how students associate spatial concepts with mathematics or geography is not merely about terminology, but about uncovering the disciplinary orientations embedded in their cognitive frameworks.

### 1.3 Interdisciplinary conceptualization of spatial concepts

This study adopts an interdisciplinary perspective to explore how learners categorize spatial concepts such as graph, coordinate, location, and area. These concepts are not exclusive to a single discipline but operate as shared resources across multiple domains. Spatial cognition, by its nature, is cross-disciplinary, and the meaning individuals attach to spatial terms is shaped by their educational exposure, contextual experiences, and cognitive predispositions (Chen et al., 2020; Vasilyeva and Lourenco, 2012). Accordingly, students’ spontaneous associations with spatial concepts can be viewed as reflective of their deeper disciplinary orientations and conceptual understandings.

### 1.4 Linking theory to method

The study’s methodological design aligns with a deductive use of theoretical frameworks. It uses pre-established disciplinary categories (mathematics, geography, both, or neither) to analyze patterns in participants’ word associations. This classification is then subjected to quantitative techniques such as Fleiss’ Kappa for inter-rater reliability, chi-square tests for distribution differences, and PCA and cluster analysis to uncover latent conceptual structures.

The framework supports this methodological strategy by justifying the use of disciplinary orientation in spatial concept associations as a core construct. Moreover, it helps interpret the results through a theoretically grounded lens that explains why individuals might favor one disciplinary frame over another when interpreting ambiguous spatial terms.

Although the framework draws on established theory, it allows flexibility for interpreting unexpected patterns. As suggested by Garvey and Jones (2021), theoretical frameworks in exploratory or mixed-methods research should not rigidly constrain analysis but should instead facilitate insight into novel or hybrid conceptualizations. In this study, the interdisciplinary nature of spatial cognition encourages openness to findings that may not fully align with disciplinary boundaries.

### 1.5 Purpose and scope of research

The purpose of this study is to investigate students’ disciplinary tendencies in relation to spatial concept associations by analyzing their conceptual responses to selected terms. Specifically, the research explores how participants cognitively link spatial concepts (Scale, Distance, Coordinate, Location, Graph, Slope, Environment, and Area) to the disciplines of mathematics, geography, both, or neither, and how these associations reflect underlying orientations and interdisciplinary thinking. The study adopts a stimulus-based associative word task, in which participants generate spontaneous word associations for each of the eight spatial stimuli. These responses are then categorized by expert raters and analyzed using a combination of descriptive, inferential, and multivariate statistical methods.

The selection of mathematics and geography as disciplinary anchors is grounded in their shared yet distinct treatment of spatial knowledge. Mathematics, situated within the STEM domain, emphasizes abstract reasoning through symbolic representations such as coordinates, graphs, geometric transformations, and numerical scaling (Mix, 2019; Geary, 2022). In contrast, geography, traditionally placed within the social sciences and humanities, operationalizes spatial understanding in relation to real-world contexts such as location, environment, landforms, and human–environment interactions (Ishikawa, 2021; Lee and Bednarz, 2012). Although both disciplines involve spatial reasoning, their epistemological orientations and curricular emphases differ, making them ideal for exploring how students perceive and categorize spatial concepts in interdisciplinary settings.

This distinction reflects broader educational structures. In many national curricula, including those of Türkiye, the United States, and the UK, mathematics is positioned as a core STEM subject, while geography appears under social studies or humanities, despite its increasing integration with geospatial technologies (Roberts, 2013; National Research Council, 2006). International assessments such as PISA also treat mathematics and geography as separate domains, although both include items requiring spatial thinking (OECD, 2018). Hence, analyzing spatial concept associations across these two disciplines offers a meaningful lens into how students cognitively navigate interdisciplinary boundaries in education. The scope of the study includes:

- Classifying participants’ associative responses based on expert-coded disciplinary categories (mathematics, geography, both, neither).
- Measuring inter-rater reliability using Fleiss’ Kappa to ensure consistency in expert judgments.
- Computing individual bias scores to quantify participants’ directional orientation toward mathematics or geography.
- Analyzing the distribution of associations across the eight common concepts using chi-square tests and standardized residual heatmaps.
- Revealing underlying conceptual structures through PCA and Ward’s hierarchical clustering.
- Examining the impact of demographic variables, including gender, mode of transportation, and self-reported subject orientation, on participants’ spatial concept associations.

This research contributes to a deeper understanding of how learners conceptually frame spatial knowledge across disciplines. It introduces an innovative, association-based method for identifying disciplinary orientation in spatial thinking and provides insights into how individual and contextual factors shape the interpretation of spatial concepts within STEM and social science boundaries.

## 2 Materials and methods

### 2.1 Research design

This study employed a quantitative, cross-sectional, and descriptive design to examine students’ disciplinary tendencies in spatial concept associations. Specifically, the research analyzed students’ associative responses to eight spatial concepts and evaluated the distribution and structure of these associations across four disciplinary categories: mathematics, geography, both, or neither. Comparable methodological approaches have been reported in recent studies investigating spatial abilities and their links to academic achievement, where cross-sectional and descriptive designs were applied to assess spatial intelligence in relation to student performance in domains such as anatomy and architecture (Berkowitz et al., 2021; Aspanani et al., 2023). This design was considered appropriate as the study aims to explore patterns and structures in students’ conceptual associations rather than to test causal relationships, making a descriptive and cross-sectional approach methodologically suitable.

### 2.2 Participants and sampling

The study sample consisted of 417 eighth-grade students enrolled in public middle schools in Antalya, Türkiye, during the 2024–2025 academic year. Of the participants, 209 were female and 208 were male, indicating a balanced gender distribution. In terms of daily school transportation, 107 students used school buses, 149 walked, and 161 traveled by private vehicle. These variables were included to examine potential contextual influences on spatial concept associations. A convenience sampling strategy was employed due to practical and logistical considerations, including accessibility to schools, administrative permissions, and the need to conduct the data collection under standardized classroom conditions. This approach enabled the collection of a large volume of associative-response data within a controlled educational setting, which is essential for ensuring consistency in stimulus administration and response generation. However, it is important to note that convenience sampling does not provide all individuals in the population with an equal probability of selection. Therefore, the sample cannot be considered statistically representative of the broader population of middle school students in Türkiye. Rather than aiming for statistical generalization, the study seeks analytical generalization by identifying patterns in students’ spatial concept associations and disciplinary orientations. Accordingly, the findings should be interpreted within the contextual boundaries of the sampled population.(Andrade, 2020; Etikan et al., 2016).

### 2.3 Data collection tool and procedure

**Figure 1:**
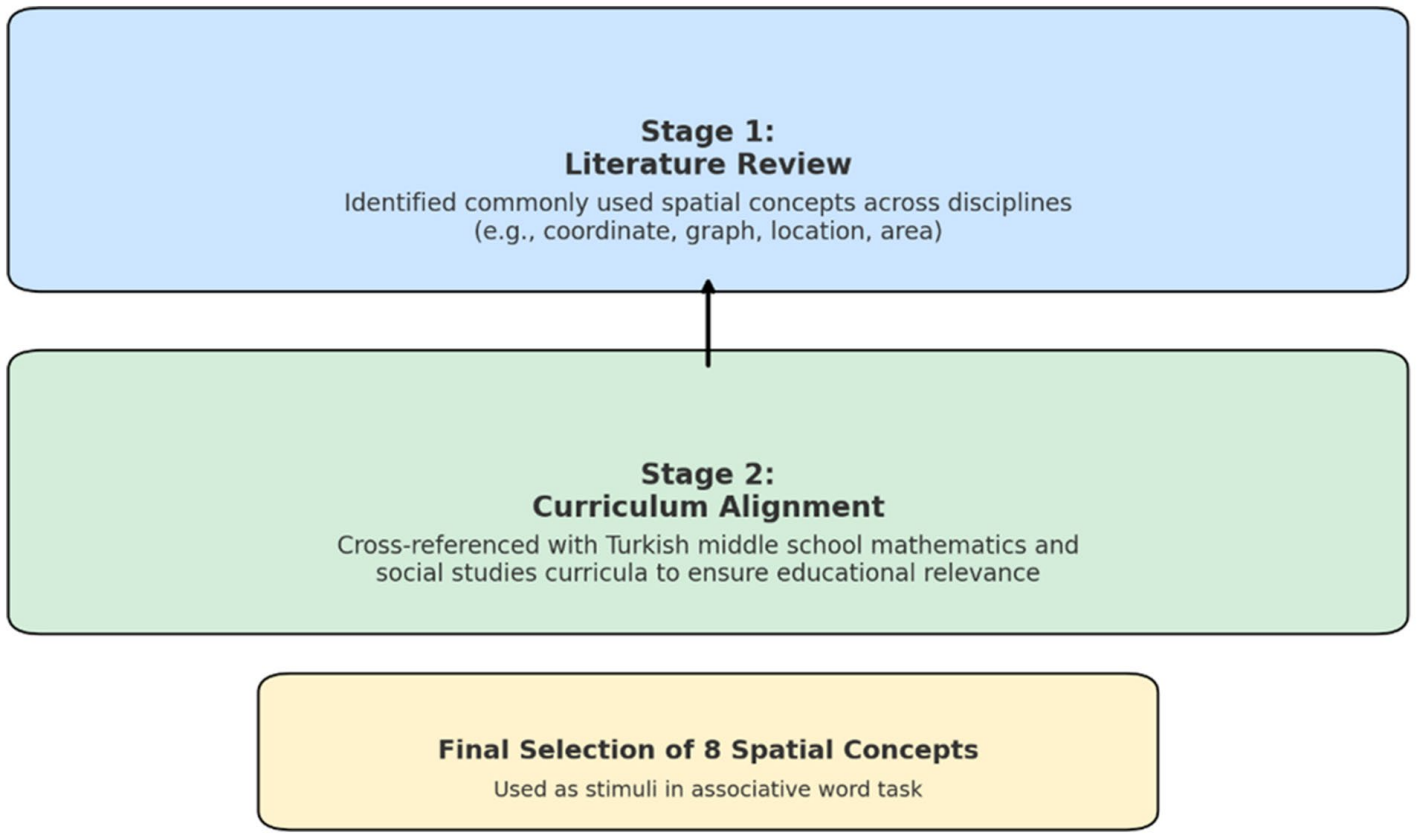
Selection of common spatial concepts.

The data for this study were collected through a stimulus-based word association task designed to elicit participants’ spontaneous conceptual responses to spatial terminology. Each participant was presented with a list of eight spatial concepts—Scale, Distance, Coordinate, Location, Graph, Slope, Environment, and Area—and was asked to generate five associative words for each stimulus. The task was administered in a supervised classroom setting to ensure procedural consistency across all participants. Selection of common spatial concepts illustrated in Figure 1.

As illustrated in Figure 1, selection of the eight common concepts was based on a two-stage process involving both international literature and national curriculum analysis. First, a focused literature review was conducted to identify spatial concepts that are commonly used across multiple academic disciplines. Studies in fields such as STEM education, geography, geoscience, music, and law demonstrate that spatial concepts are interpreted and applied differently depending on disciplinary context (Kuhn, 2012; Atit et al., 2020; McNeal and Petcovic, 2020; Jo and Hong-Dwyer, 2024; Ziemer, 2020; Bennett and Layard, 2015). Based on these findings, a preliminary list of widely recognized and disciplinary flexible spatial concepts was established.

In the second stage, this list was cross-referenced with the middle school mathematics and social studies curricula in Türkiye to ensure that the concepts were not only theoretically relevant but also pedagogically appropriate for the target student population. This dual alignment with both international literature and national education standards provided a strong basis for the inclusion of these eight spatial concepts in the associative word task used in the study.

To ensure the developmental appropriateness and conceptual balance of stimuli, the list of spatial concepts was reviewed by a panel of four domain experts, two in mathematics education and two in social studies education. Experts confirmed that each word was sufficiently general, conceptually clear, and capable of eliciting interdisciplinary associations without introducing ambiguity, while remaining accessible and pedagogically appropriate for the target group of eighth-grade students. This process provided an expert opinion foundation supporting the content appropriateness and surface-level clarity of the prompts used within the assessment tool.

To further strengthen the validity of the stimulus set, the selection process was framed within a content validity approach, ensuring that the included concepts adequately represent the domain of spatial thinking across disciplines. In addition, the clarity of the stimuli was supported by presenting them as single, commonly used terms rather than complex or technical definitions, thereby minimizing the risk of misinterpretation and allowing participants to generate spontaneous and unbiased associations. This approach is consistent with established practices in associative-response methodologies, where overly complex or leading prompts may constrain cognitive responses. Furthermore, standardized administration procedures and uniform instructions ensured that all participants interpreted the stimuli under comparable conditions, supporting procedural consistency and interpretive reliability. Taken together, these procedures ensure that the stimulus set demonstrates content validity by adequately representing interdisciplinary spatial constructs, while also maintaining conceptual clarity and interpretability for the target population. The use of simple, familiar terms and expert validation reduces the likelihood of ambiguity, increasing confidence that participants’ responses reflect their underlying conceptual associations rather than misinterpretations of the stimuli.

The responses obtained from participants were subjected to a data cleaning process prior to analysis. During this process, all responses were first reviewed; duplicate statements, responses with the same meaning but differing spelling, blank fields, and incomprehensible statements were checked. Following this process, a list of unique response terms was prepared for use in expert coding. As a result, a total of 1,827 unique responses were included in the coding process. The final category assignments were determined through a consensus meeting among the experts.

Each unique statement was independently classified by subject matter experts into four categories: Mathematics (M), Geography (G), Both Fields (B), and Neither (N). The Mathematics category includes statements primarily corresponding to mathematical concepts, operations, or representations; the Geography category includes statements related to the spatial environment, direction, maps, location, or geographical context; the Both Fields category includes statements that can be meaningfully associated with both mathematics and geography; and the None category encompasses responses that cannot be reasonably associated with either of these disciplines. Four experts independently coded the associative responses, and to assess the reliability of their judgments, Fleiss’ Kappa coefficient was calculated to quantify inter-rater reliability, providing statistical evidence of the consistency of the coding procedure across independent expert evaluations. The results of this analysis are presented in Table 1.

**Table 1:** Agreement percentage of experts for the concepts.

Agreement percentage	Chance percentage	Fleiss Kappa coefficient	*p*	%95 confidence
0.823	0.250	0.764	0.001	[0.746;0.780]

The coding procedure followed a structured multi-step protocol to ensure consistency and transparency. First, all unique responses were compiled into a standardized coding list after data cleaning. Each response was then independently evaluated by four experts based on predefined category definitions (Mathematics, Geography, Both, Neither). Coding decisions were guided by the primary semantic meaning of each response within its contextual usage.

Following independent coding, discrepancies among raters were identified and resolved through a consensus meeting, during which experts discussed ambiguous cases and agreed on final category assignments. This process ensured that all classifications reflected shared expert judgment rather than individual interpretation.

To further support methodological rigor, the coding scheme was applied consistently across all responses, and no iterative recoding or category modification was performed after the initial classification framework was established. This approach enhances the replicability and reliability of the coding process.

Prior to the experiment, all students received the same standardized training. Students were asked to write down the first five words that came to mind for each stimulus concept, to respond individually, and not to discuss their answers with peers during the task. Since the aim was to capture spontaneous conceptual associations, no feedback regarding accuracy was provided during the task. The task was completed in approximately 10 min in a normal classroom setting under the researcher’s supervision.

### 2.4 Assessment of inter-rater reliability among expert coders: Fleiss’ Kappa results

Eight stimulus concepts were used to elicit disciplinary associations for spatial concepts. Domain experts evaluated the associative words provided by participants in relation to each stimulus word, categorizing them into four disciplinary groups: M = mathematics, G = geography, B = both disciplines, and N = neither discipline. Four experts independently coded the associative responses, and to assess the reliability of their judgments, the Fleiss’ Kappa coefficient was calculated. The results of this analysis are presented in Table 1.

Table 1 shows that the percentage agreement among expert raters for the classification of words was 82.3%, while the expected agreement by chance was 25.0%. The Fleiss’ Kappa coefficient was calculated as 0.764. According to the interpretive guidelines provided by Altman (1999) and Landis and Koch (1977), values below 0 indicate very poor agreement; values between 0.01 and 0.20 represent slight agreement; 0.21–0.40, fair agreement; 0.41–0.60, moderate agreement; 0.61–0.80, substantial agreement; and 0.81–1.00, almost perfect agreement. Based on this classification, the inter-rater agreement in the present study was found to be substantial.

### 2.5 Distribution of spatial concept association tendencies by disciplinary domain: classification based on bias scores

To determine individuals’ tendencies based on the words they generated, the following formula was used:

$$\text{bias}=\frac{\left(M_{i}+0.5B_{i})-(G_{i}+0.5B_{i}\right)}{M_{i}+G_{i}+B_{i}+N_{i}}$$

The bias score used in this study is a normalized directional difference index designed to summarize the relative dominance of participants’ mathematics- and geography-oriented associations. The primary aim of the difference score approach is to reduce the directional distinction between two components to a single interpretable indicator; however, such scores should be treated not as direct psychometric scale scores but as derived indicators defined according to the research objective (Bedeian and Day, 1994). In this regard, the current formula standardizes the difference between mathematics- and geography-oriented associations by dividing it by the total number of associations, thereby reducing the influence of differences in total response production among individuals. Positive values of the resulting score indicate a mathematics orientation, negative values indicate a geography orientation, and values close to zero indicate a balanced or indistinct orientation. Therefore, the bias score has been interpreted not as a psychometric score measuring a latent trait level, but as a summary index representing the relative direction in the participant’s association profile. The methodological literature on difference scores recommends that the theoretical clarity and interpretive limitations of such derived indicators be clearly stated; in the present study, the score was used within this framework as a classificatory and comparative indicator (Rogosa and Willett, 1983). The validity of the bias score in this study was addressed not in terms of a scale score, but through its ability to consistently and interpretably summarize the relative direction of the disciplinary classification based on expert coding at the participant level.

To ensure the robustness of this measure, the bias score was conceptualized as a normalized directional index rather than a latent psychometric scale. Its primary function is to capture relative disciplinary orientation by balancing mathematics- and geography-related associations within individuals. The normalization by total response count reduces the influence of individual differences in response production, thereby enhancing comparability across participants.

Although the bias score does not represent a standardized scale with established construct validity, its interpretive strength lies in its consistency with the underlying categorical coding framework and its alignment with the study’s analytical purpose. Similar difference-based indices have been used in prior research to summarize directional tendencies in categorical data.

Furthermore, the robustness of the measure is supported indirectly through the reliability of the coding process (as indicated by Fleiss’ Kappa) and the convergence of results across multiple analytical techniques, including chi-square tests, PCA, clustering, and non-parametric comparisons. These converging findings provide additional confidence that the bias score captures meaningful patterns rather than random variation.

### 2.6 Data analysis

Data analysis was carried out using both descriptive and multivariate statistical techniques. To determine the directional disciplinary tendencies of each participant, bias scores were calculated by comparing the frequency of associations related to mathematics and geography, with shared associations weighted accordingly. To examine whether the distribution of disciplinary associations varied significantly across the eight common concepts chi-square tests were employed.

To further explore these differences, standardized and adjusted residuals were computed, and the results were visualized using a heatmap, which allowed for the identification of categories with statistically meaningful over- or under-representation.

Principal component analysis (PCA) was applied to holistically examine the distribution patterns of the eight stimulus concepts across four categories (Mathematics, Geography, Both Fields, Neither). In this study, PCA was used not to establish an implicit measurement model, but to summarize the fundamental axes of variation among the stimulus category profiles and to represent the multivariate pattern in fewer dimensions. In the analysis, the category profiles of the stimuli were treated as a numerical data matrix; in interpreting the components, eigenvalues, the scree plot, the proportion of total variance explained, and the magnitude of component loadings were considered together. In interpreting the loadings, coefficients with an absolute value of 0.40 or higher were considered significant. When deciding on the number of components, the criterion of eigenvalue >1 was evaluated as an auxiliary indicator; however, the primary decision was made by considering the appearance of the scree plot and the significance of the explained variance. Since PCA is an exploratory method based on summarizing the linear interrelationships among variables, it was deemed appropriate in this study for visualizing and interpreting the structural separation of stimulus profiles (Jolliffe, 2002).

Following PCA, the Ward hierarchical clustering method was applied to group stimuli with similar association profiles. The Ward method is based on the minimum variance approach, which aims to minimize the increase in intra-cluster variance as clusters are merged. Therefore, it is a suitable choice for obtaining compact and interpretable clusters in data structures consisting of a small number of stimuli that are intended to be interpreted based on profile similarity (Ward, 1963; Murtagh and Legendre, 2014). The distance metric used in the clustering analysis was set to squared Euclidean. Thus, the dimensionality reduction obtained via PCA was complemented by hierarchical clustering to examine whether similar features were grouped within the same structure.

In the data analysis process, bias scores summarizing each participant’s disciplinary orientation were first calculated. These scores enabled comparisons between individuals by standardizing the relative difference between mathematics- and geography-oriented associations relative to the total number of associations. Preliminary inspection indicated that the distribution of bias scores did not fully meet parametric assumptions; therefore, nonparametric tests were preferred.

Nonparametric tests were used to examine whether participants’ spatial concept association scores differed according to demographic variables. Since bias scores produce values within a limited range and serve as a derived orientation index, ranking-based methods were preferred over parametric tests in group comparisons. This choice provides a more cautious analytical approach in light of the possibility that the distribution characteristics of the scores may not fully meet parametric assumptions. In this context, the Mann–Whitney *U* test was applied for comparisons by gender, while the Kruskal–Wallis *H* test was used for comparisons based on mode of transportation to school and self-reported field orientation. Where necessary, effect sizes were calculated using the *r* and *ε*^2^ coefficients, respectively.

## 3 Results

**Figure 2:**
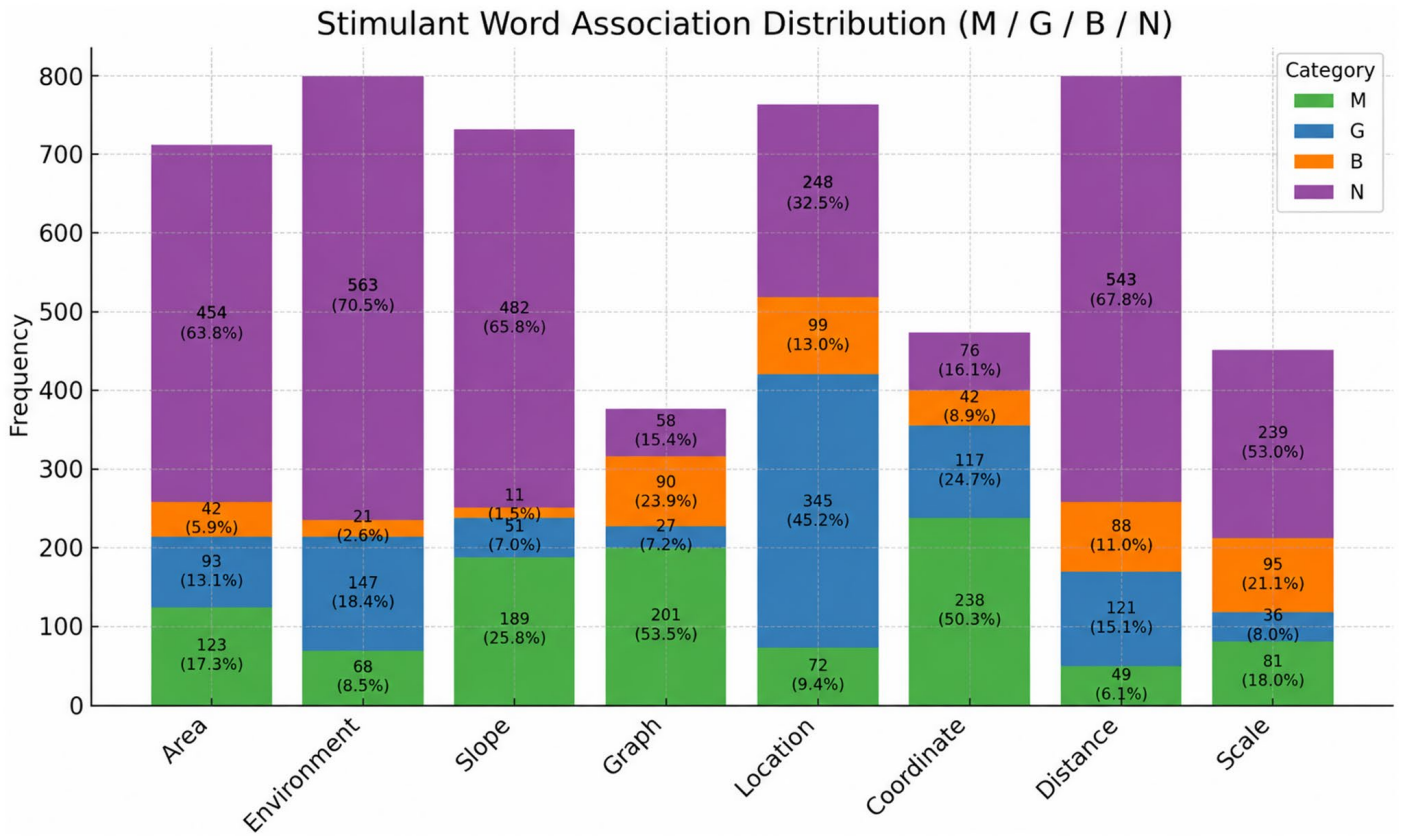
Distribution of participant-generated associative words across eight spatial stimulus concepts categorized by disciplinary orientation.

Each participant generated five associative words for each stimulus word. In total, participants produced 16,680 associative words, of which 1,827 were unique entries. Number of associative words produced by participants are illustrated in Figure 2.

### 3.1 Number of associative words produced by participants and frequency analysis

Distribution of participant-generated associative words across eight spatial stimulus concepts categorized by disciplinary orientation presented in Figure 2.

Figure 2 presents the distribution of participant-generated associative words across eight spatial stimulus concepts categorized according to four disciplinary orientations: Mathematics (M), Geography (C), Both (B), and Neither (N). The total number and percentage of associations falling into each category are displayed for each stimulus word, allowing for comparative analysis of participants’ implicit disciplinary conceptualizations.

The analysis reveals that certain stimulus words elicited a disproportionately high number of associations falling into the “N” category, indicating a lack of clear disciplinary anchoring. Notably, Environment (70.5%), Slope (65.8%), Distance (67.8%), and Area (63.8%) were most frequently associated with words classified outside the predefined disciplinary frameworks. This pattern suggests that these concepts, while spatial in nature, may be perceived by students as more context-dependent or less conceptually integrated within formal academic domains.

Conversely, words such as Coordinate (50.3%), Location (53.5%), and Graph (23.9%) demonstrated a more balanced disciplinary distribution. Specifically, Location had the highest percentage of associations categorized under “Both” (45.2%), followed by Graph (23.9%) and Coordinate (16.1%), indicating that these concepts hold potential as interdisciplinary connectors between mathematics and geography.

The “M” category was most prominent for Slope (25.8%), Area (17.3%), and Scale (18.0%), suggesting a stronger mathematical orientation in students’ mental representations of these concepts. In contrast, Coordinate (24.7%), Distance (15.1%), and Location (13.0%) elicited relatively more geography-aligned associations.

### 3.2 Word cloud of participants’ associative responses

**Figure 3:**
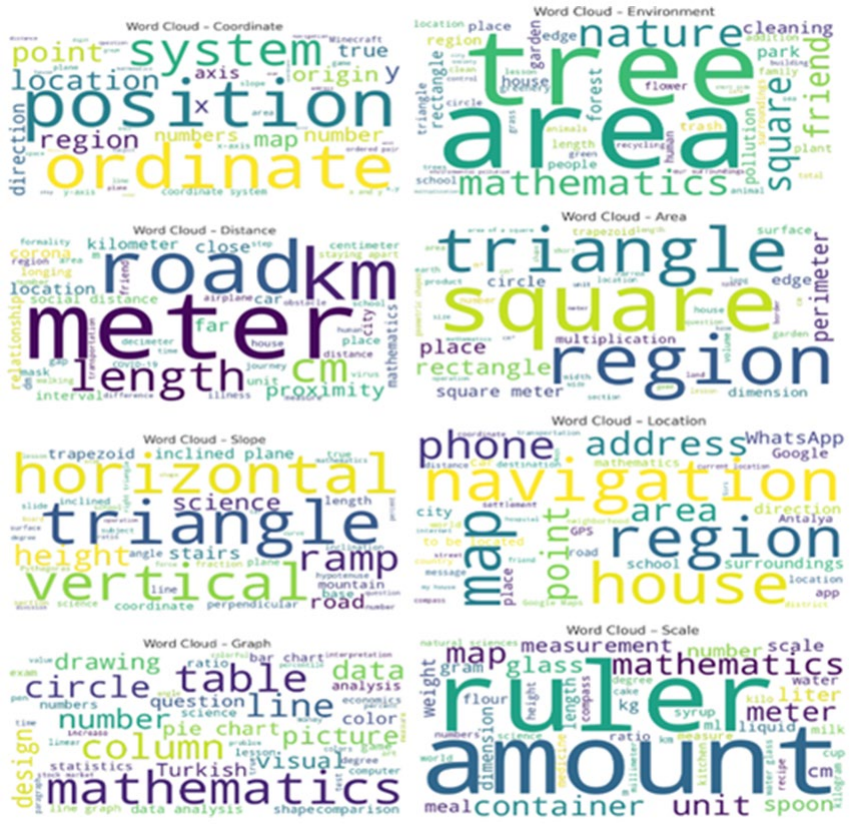
Word clouds displaying the 50 most frequent associative words generated by participants for each stimulus concept.

Word clouds displaying the 50 most frequent associative words generated by participants for each stimulus concept presented in Figure 3.

### 3.3 Statistical comparison of association categories: chi-square test and Cramér’s *V* results

**Figure 4:**
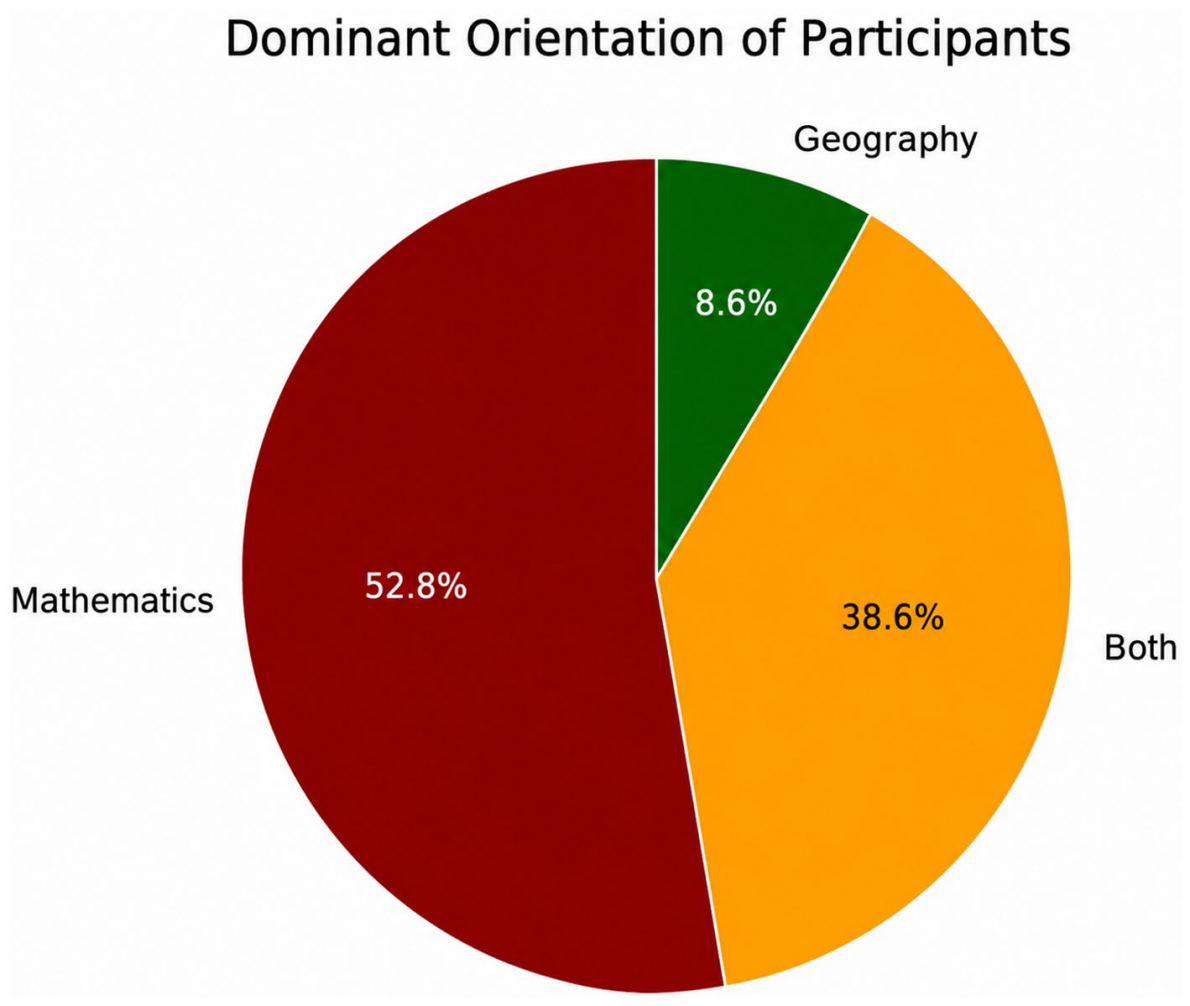
Distribution of participants’ dominant disciplinary orientation based on associative word bias scores.

According to the analysis, participants’ tendencies based on their associative words are presented in Figure 4.

As seen on Figure 4, it was found that 52.8% of the participants demonstrated a tendency toward mathematics, 38.6% reflected an equal consideration of both fields, while 8.6% showed a tendency toward geography. To examine the distribution of associative words across the four disciplinary categories [Mathematics (M), Geography (C), Both (B), and Neither (N)], a chi-square test was conducted. The results indicated that the distributions differed significantly across the eight stimulus words, *χ*^2^(21) = 1685.6, *p* < 0.001, with a Cramér’s *V* value of 0.190. This global test demonstrates that the M/C/B/N distributions for the eight stimuli were not random and that the overall effect size was moderate (*V* ≈ 0.19).

To identify significant cells within the resulting contingency tables, standardized and adjusted residual values were analyzed following the chi-square test. This analytic procedure is consistent with recommended practices for interpreting cell-level contributions in categorical data (Shan and Gerstenberger, 2017). The disciplinary associations varied across each stimulus word. To further explore the domain-specific tendencies embedded in these associations, a heatmap of standardized residuals was examined.

### 3.4 Heatmap analysis illustrating differences in disciplinary associations

**Figure 5:**
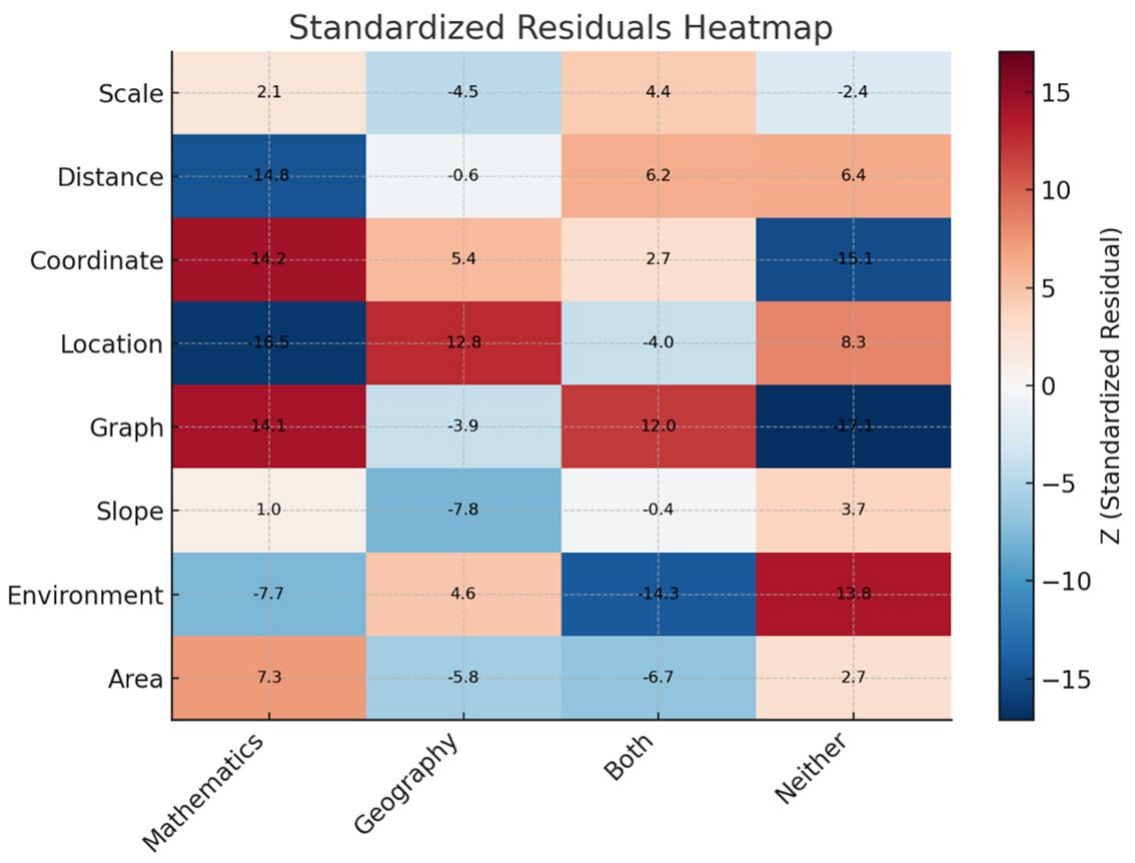
Standardized residual heatmap of stimulus word associations by disciplinary category.

Standardized residual heatmap of stimulus word associations by disciplinary category is presented in Figure 5.

To examine the disciplinary tendencies of the associative words, a standardized residuals heatmap was analyzed (Figure 5). The results indicated that the words distance, location, and environment generated the fewest mathematics-related associations, whereas coordinate, graph, and area elicited the highest number of associations with mathematics. In contrast, the words that yielded the fewest associations with geography were slope and area, while location and coordinate were the most frequently associated with geography. In terms of dual associations distance and graph emerged as the most common, whereas environment and area were the least frequently associated with both disciplines. Regarding associations falling outside of both disciplinary domains, environment, location, and distance were the most prevalent, whereas graph and coordinate had the fewest associations in this category.

To gain a holistic understanding of the categorical proportions and interrelations among the eight stimulus words, a two-step multivariate analysis was conducted. First, PCA was employed to uncover the underlying dimensions of variation within the associative word data. Following this, Ward’s hierarchical clustering method was applied to group stimulus words exhibiting similar associative profiles. This dual analytical strategy aligns with established approaches for dimensional reduction and structured clustering in multivariate data analysis (Chou and Wang, 2010; Randriamihamison et al., 2021).

### 3.5 Identification of conceptual dimensions through PCA

**Figure 6:**
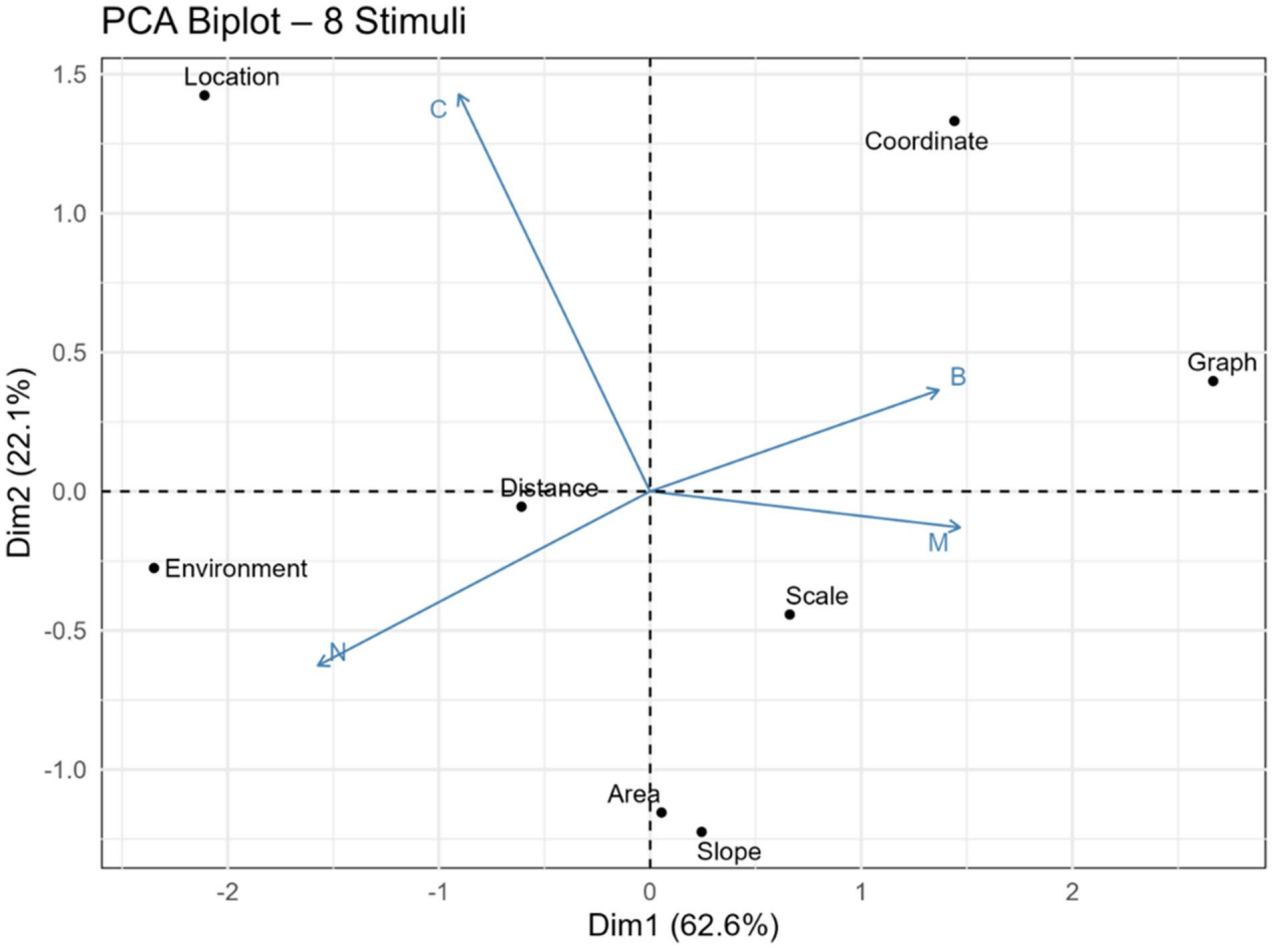
PCA biplot of stimulus words and disciplinary orientations.

PCA biplot of stimulus words and disciplinary orientations presented in Figure 6.

PCA based on the categorical frequencies of the eight stimulus words (Area, Environment, Slope, Graph, Location, Coordinate, Distance, Scale) revealed two principal components. The first dimension (Dim1) accounted for 62.6% of the total variance, while the second dimension (Dim2) explained an additional 22.1%, together capturing 84.7% of the total variation in the dataset (Figure 6).

Dim1 primarily differentiates between mathematics- and geography-oriented associations. Specifically, positive values on Dim1 correspond to concepts predominantly associated with mathematics or both disciplines, whereas negative values reflect associations with geography or with neither discipline. Accordingly, the words Area, Slope, and Scale exhibited strong positive loadings, indicating dominant mathematical associations. Coordinate and Graph were situated near the center of Dim1, reflecting high association with both disciplines. In contrast, Location exhibited strong negative loadings, suggesting a clear geographical orientation, while Environment was closely aligned with associations falling outside of both disciplinary categories.

Dim2, on the other hand, distinguishes between dual-discipline or geography associations (positive values) and mathematics or non-disciplinary associations (negative values). For example, Graph and Distance were positioned centrally, reflecting dual associations; Location and Coordinate leaned toward geography; Environment aligned with the non-disciplinary category; and Scale, Area, and Slope clustered toward mathematics.

To further explore the conceptual clustering of these stimulus words, Ward’s hierarchical clustering method was employed. This analysis not only grouped similar associative profiles but also provided insight into the cognitive and disciplinary dimensions underlying these clusters. Given Ward’s emphasis on minimizing within-cluster variance, the method was particularly effective in identifying conceptually coherent groups reflective of mathematics- and geography-related cognitive patterns. The clustering conducted on the PCA-derived dimensions enabled the identification of latent structures within the dataset that are pedagogically and theoretically meaningful.

### 3.6 Identification of disciplinary association profiles through hierarchical clustering

**Figure 7:**
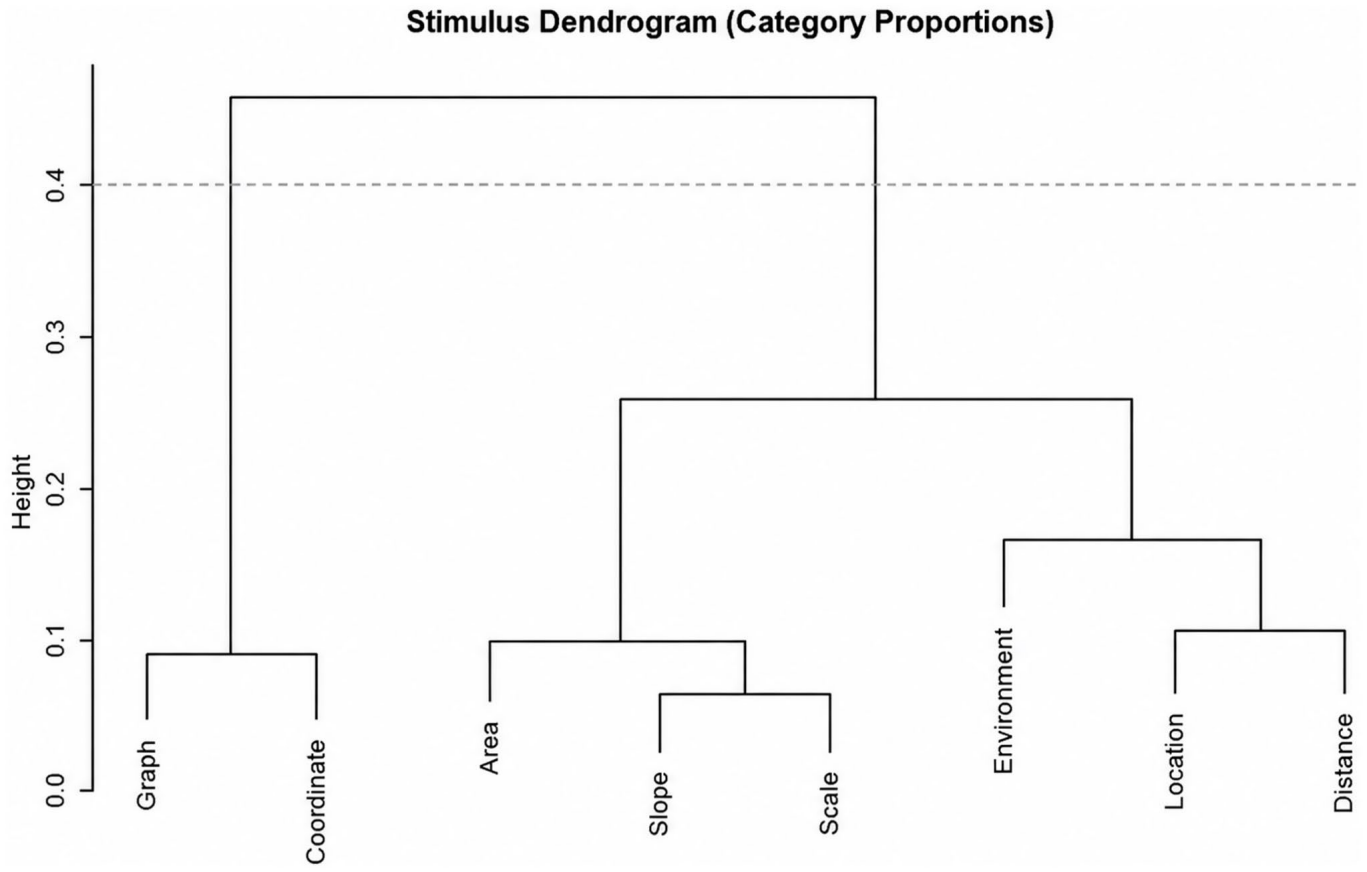
Hierarchical clustering dendrogram of stimulus words based on category proportions.

Hierarchical clustering dendrogram of stimulus words based on category proportions presented in Figure 7.

As in the PCA results, the clustering analysis revealed three distinct disciplinary groupings (Figure 7). The words graph and coordinate, both of which elicited strong associations with both mathematics and geography, formed the first cluster. The second cluster included area, slope, and scale, which were predominantly associated with mathematical concepts. The third cluster, consisting of location, distance, and environment, reflected associations that were more spatial-contextual or environmentally oriented, suggesting a stronger connection to geographic thinking.

This clustering structure indicates that the associative loadings of words tend to align with specific disciplinary domains. Words in the first cluster appear to serve as shared cognitive representations between mathematics and geography, suggesting a conceptual bridge between the two fields. The second cluster includes words more tightly linked to mathematical constructs, while the third encompasses concepts that evoke contextual, place-based, or environmental associations.

Ward’s hierarchical clustering method effectively defined this separation by maximizing intra-cluster homogeneity and inter-cluster heterogeneity. The resulting clusters not only reflect frequency-based differences but also provide conceptually meaningful patterns in terms of disciplinary alignment. These findings are significant for identifying points of convergence and divergence in participants’ conceptual representations across academic disciplines.

In the analysis of gender-based differences in spatial concept associations, the Mann–Whitney *U* test was applied. The effect size for this test was interpreted using the rank-biserial correlation coefficient, with values defined as follows: small effect (0.10 ≤ *r* < 0.30), medium effect (0.30 ≤ *r* < 0.50), and large effect (*r* ≥ 0.50), as suggested by Willson (1976). For the Kruskal–Wallis *H* test, the effect size was calculated using epsilon squared (*ε*^2^), interpreted as small (0.01), medium (0.06), and large (0.14), according to Cohen (2013). The results of the Mann–Whitney *U* test examining whether spatial concept association scores differed by gender are presented in Table 2.

**Table 2:** Gender-based differences in spatial concept association levels.

Scale	Sex	*n*	Mean rank	Sum of ranks	*U*	*z*	*p*	Rank-biserial correlation
Spatial concept association score	Female	209	130,74	27,324,00	5,379,000	−13,319	0.000	0.653
	Male	208	287,64	59,829,00				

### 3.7 Gender-based differences in spatial concept association levels

An examination of the Table 2 reveals a statistically significant difference in spatial concept association scores between female and male participants (z = −13.319; *p* = 0.001 < *α* = 0.05). Analysis of the mean ranks indicates that the male group had a significantly higher mean rank than the female group. The effect size is considered large.

### 3.8 Differences in spatial concept association levels by mode of transportation

An examination of Table 3 reveals that there is a statistically significant difference in spatial concept association scores based on participants’ mode of transportation [*H*(2) = 367.516; *p* value = 0.001 < α = 0.05]. When the mean ranks across the transportation groups are analyzed, it is observed that participants who commute by school bus have significantly higher mean ranks than those who commute by walking or by their own vehicle. Furthermore, individuals who commute using their own vehicle exhibit significantly higher mean ranks compared to those who commute on foot. The effect size is considered high.

**Table 3:** Mode of transportation-based differences in spatial concept association levels.

Scale	Mode of transportation	*n*	Mean Rank	Kruskall Wallis-*H*	df	*p*	Significant Difference	$\varepsilon^{2}$
Spatial concept association score	School bus (1)	107	364,00	367,516	2	0.001	1 > 2	0.883
							1 > 3	
							3 > 2	
	Walking (2)	149	75,00					
	Family car (3)	161	230,00					

### 3.9 Differences in spatial concept association scores by disciplinary orientation

When Table 4 is examined, a statistically significant difference was found in students’ spatial concept association scores across the disciplinary orientation groups [*H*(2) = 7.941, *p* = 0.019 < *α* = 0.05]. *Post-hoc* analysis of mean ranks revealed that the mathematics-oriented group had significantly higher mean ranks compared to the dual-orientation group. The effect size was found to be small.

**Table 4:** Differences in spatial concept association scores by disciplinary orientation based on Kruskal–Wallis *H* test.

Scale	Disciplinary orientation	*n*	Mean rank	Kruskall Wallis-*H*	df	*p*	Significant difference	$\varepsilon^{2}$
Spatial concept association score	Geography (1)	36	210,08	7,941	2	0.019	Mathematics > Both	0.014
	Both (2)	161	188,60					
	Mathematics (3)	220	223,75					

## 4 Results and discussion

This study examined how middle school students’ associative responses to spatial stimuli align with disciplinary orientations (mathematics, geography, both, or neither) and analyzed the cognitive and conceptual structures that organize these associations. To ensure the validity of subsequent analyses, it was first necessary to confirm the reliability of the categorical classification of associative words.

Four domain experts independently coded each associative response according to the four predefined categories. To evaluate the consistency of their classifications, Fleiss’ Kappa coefficient was calculated. Given that Fleiss’ Kappa is appropriate for measuring inter-rater reliability among more than two raters working with categorical data (Park and Kim, 2015), its application here was methodologically warranted. The results revealed a statistically significant and substantial level of agreement among coders, indicating that the classification scheme was applied consistently across raters. This high level of inter-rater reliability enhances the credibility of the categorization process and provides a robust foundation for the disciplinary analyses that follow (Mcdonald et al., 2019; Park and Kim, 2015).

The analysis of participant-generated associations demonstrates that students’ interpretations of spatial concepts vary considerably in their disciplinary orientation. The predominance of “Neither” categorizations for Environment, Slope, Distance, and Area suggests that these concepts are frequently understood outside formal disciplinary boundaries. Such outcomes indicate that many students conceptualize these concepts in general or everyday contexts rather than as structured academic constructs. This is particularly evident in place-based or community-centered understandings of space, as demonstrated by Jones (2021), who found that urban youth interpreted land, ownership, and spatial justice through local experiences such as redlining and benevolent societies. This aligns with findings in the literature suggesting that certain spatial concepts, particularly those encountered in informal settings or without explicit curricular integration, may not be strongly anchored within mathematics or geography domains (Cai et al., 2020; Schlemper et al., 2018; W. Zhang et al., 2023). The ambiguous status of Environment is especially notable, as it is a term that spans multiple disciplines but may lack precise conceptual framing in students’ cognitive schemas.

In contrast, the concepts Location, Graph, and Coordinate were more frequently associated with both mathematics and geography, indicating their interdisciplinary salience. The relatively high percentages of “Both” responses, particularly the 45.2% for Location, suggest that these concepts function as cognitive connectors across subject areas. These findings are consistent with prior research emphasizing the dual role of certain spatial concepts in mathematical reasoning and geographic thinking (Cai et al., 2020). For instance, Coordinate systems underpin both algebraic graphing and geographic positioning, while Location is fundamental in spatial reasoning across curricula.

Distinct disciplinary anchoring was evident for several spatial concepts. Slope, Scale, and Area were most frequently associated with mathematics, likely due to their foundational role in the teaching of geometry, measurement, and proportional reasoning. This pattern may also be explained by the structure of mathematics and geography instruction in the Turkish educational context, where mathematics curricula emphasize formalized spatial reasoning through symbolic and problem-based tasks, whereas geography instruction tends to prioritize descriptive and contextual knowledge. These concepts are commonly introduced through formulaic problem-solving tasks and are emphasized in mathematics curricula, particularly in middle school education, where the focus is on abstract reasoning and quantitative analysis. Recent evidence from Chinese elementary students also shows that spatial reasoning constructs such as mental rotation, visualization, and orientation significantly predict mathematical performance across number, geometry, and data display domains, reinforcing the strong disciplinary link between mathematics and spatial cognition (Xu et al., 2025). In contrast, Coordinate, Distance, and Location elicited stronger associations with geography, highlighting their relevance in spatial navigation, mapping, and geographic information systems. Such divergence in disciplinary association reflects how educational experiences and curriculum design guide students’ conceptual development.

This pattern is well-supported in literature. Grigoroglou and Papafragou (2019) argue that the meaning and use of spatial concepts are context-dependent, shaped by both semantic framing and the practical applications emphasized within disciplinary instruction. In mathematics, concepts such as slope and area are often introduced as part of formal systems governed by precision and formulae, while in geography, concepts like coordinate and location are taught in relation to real-world spatial relationships and environmental contexts. The divergent instructional strategies employed across these domains reinforce domain-specific interpretations of shared spatial vocabulary. Over time, such reinforcement leads students to anchor certain concepts more firmly within one discipline, even if those concepts are theoretically applicable across multiple domains.

The statistically significant result of the chi-square test indicates that the distribution of participant-generated associative words across the disciplinary categories is not uniform across the eight spatial stimulus words. This confirms that students’ associations are meaningfully structured and vary depending on the specific spatial term in question. The observed chi-square value [*χ*^2^(21) = 1685.6, *p* < 0.001] suggests a strong deviation from what would be expected under a model of independence, implying that participants do not associate spatial concepts with academic disciplines in a random or undifferentiated manner.

The effect size measure, Cramér’s *V* = 0.190, further supports the presence of a moderate relationship between the type of spatial stimulus and the disciplinary orientation of the associated word. As emphasized in methodological literature, this level of association is typical in studies involving complex categorical data and indicates a meaningful, though not overwhelming, dependency between variables (McHugh, 2013). In educational contexts, such an effect size suggests that while stimulus words play a notable role in shaping disciplinary associations, additional factors likely also contribute.

Beyond the global significance of the test, the analysis of standardized and adjusted residuals provides important insights into the specific disciplinary profiles of individual stimulus words. This residual-based follow-up is essential for identifying the unique contributions of concepts to the overall statistical pattern. The finding that each spatial word exhibited a distinctive pattern of associations underscores the cognitive complexity of spatial language and its differential integration into students’ academic knowledge frameworks. Such patterns, as Franke et al. (2012) argue, reveal the specific sources of variation within categorical datasets and are vital for moving beyond omnibus statistics toward meaningful educational interpretation.

The standardized residuals analysis revealed clear disciplinary tendencies across the eight stimulus words. Concepts such as distance, location, and environment were the least associated with mathematics, consistent with their broader, everyday usage and foundational role in geography (Kuhn, 2012; Grossner et al., 2017). In contrast, coordinate, graph, and area showed strong associations with mathematics, reflecting their formal integration into mathematical curricula, particularly in geometry and data representation.

Conversely, slope and area were rarely associated with geography, suggesting their limited curricular emphasis in that domain. Location and coordinate, on the other hand, were strongly linked to geography, supporting previous findings that these are core spatial concepts in geographic instruction and GIS applications (Kuhn, 2012; Goodchild and Janelle, 2010).

Some concepts, notably distance and graph, exhibited dual associations with both disciplines, highlighting their interdisciplinary character. However, environment, location, and distance were frequently placed in the “Neither” category, suggesting a broader semantic range and less defined disciplinary anchoring. These findings underscore the flexible and context-dependent nature of spatial language among students (Grossner et al., 2017).

The PCA identified two core dimensions that collectively accounted for 84.7% of the total variance in students’ disciplinary associations with spatial concepts, highlighting the technique’s utility in uncovering latent structures within categorical datasets (Harris et al., 2011; Zhang et al., 2021). The first component (Dim1), which explained 62.6% of the variance, represented a continuum ranging from mathematics-oriented to geography-oriented associations. Spatial concepts such as Area, Slope, and Scale exhibited strong positive loadings on this axis, reflecting their established alignment with mathematical reasoning and their frequent treatment in geometry and measurement instruction. In contrast, Location loaded negatively, indicating its conceptual anchoring within geographic frameworks and its central role in spatial thinking and place-based understanding (Kuhn, 2012; Grossner et al., 2017).

The positioning of Coordinate and Graph near the center of Dim1 supports their interdisciplinary nature, as these concepts frequently appear in both mathematics and geography curricula (Harris et al., 2011; Goodchild and Janelle, 2010). The term Environment, with weak or neutral loadings, again emerged as conceptually unanchored within disciplinary boundaries, aligning with prior findings about its general or contextual interpretation. Such concepts often gain meaning through experiential and place-based learning rather than disciplinary instruction (Mitchell and Elwood, 2012).

The second component (Dim2), explaining 22.1% of the variance, further refined the conceptual structure by distinguishing between geography/dual-disciplinary associations and mathematics/non-disciplinary ones. This additional axis captured more nuanced conceptual groupings. For example, while Scale, Area, and Slope reinforced their mathematical anchoring on the negative side of Dim2, Location and Coordinate leaned positively, highlighting their geographical salience. Such dual-axis interpretations reflect PCA’s capacity to uncover complex conceptual overlaps and divergences in spatial language (Shahdoosti and Ghassemian, 2016; Yan et al., 2021).

The application of Ward’s hierarchical clustering following PCA enriched the analysis by organizing spatial concepts into coherent conceptual groupings. These clusters closely mirrored the principal component dimensions, reinforcing disciplinary and interdisciplinary associations. By minimizing intra-cluster variance, Ward’s method uncovered latent cognitive structures, offering insights into how students mentally categorize spatial concepts in relation to mathematics, geography, or both. This clustering approach not only enhances interpretability but also provides a valuable basis for designing pedagogical strategies that support disciplinary integration (Harris et al., 2011).

Ward’s hierarchical clustering to explore the cognitive structuring of spatial vocabulary provides valuable insight into how students mentally organize disciplinary associations. The emergence of three conceptually coherent clusters highlights distinct disciplinary orientations and underscores learners’ implicit grouping tendencies. This pattern aligns with the established role of Ward’s method in minimizing within-group variance and revealing semantically or cognitively coherent structures (Chavent et al., 2018). The dual-disciplinary positioning of Graph and Coordinate suggests that students recognize their shared relevance across mathematics and geography, supporting previous findings in semantic clustering and interdisciplinary cognition (Majewska et al., 2021). In contrast, the mathematical clustering of Area, Slope, and Scale reflects their dominance in mathematical instruction, particularly in geometry and measurement, whereas the third cluster signals the broader, context-dependent use of concepts like Location, Distance, and Environment (Cabezas et al., 2023).

The close correspondence between these clusters and the PCA dimensions reinforces the robustness of the findings. Using PCA to identify principal axes of variation, followed by clustering to structure categorical associations, is a well-supported analytic strategy for interpreting complex associative data (Cabezas et al., 2023; Chavent et al., 2018). Importantly, this analytic approach also contributes to the broader methodological agenda in spatial reasoning research, where scholars have underscored the lack of reliable, valid, and accessible assessments of spatial skills (Uttal et al., 2024). Recent psychometric modeling work further demonstrates that item-level characteristics exert substantial effects on task difficulty, reinforcing the value of diversified methodological approaches such as the associative-response design employed in this study (Shi et al., 2023).

The observed gender-based difference in spatial concept association scores, where male participants significantly outperformed their female counterparts, warrants careful interpretation within the broader context of existing research. The Mann–Whitney *U* test revealed a highly significant result (*z* = −13.319, *p* < 0.001) with a large effect size, suggesting that this disparity is not only statistically robust but also practically meaningful within the study sample. However, these results do not exist in isolation and must be considered alongside a body of literature that offers a more complex and sometimes contradictory picture. Previous studies have produced mixed results regarding gender differences in spatial intelligence. For example, Murray (2016) identified a moderate gender difference favoring males among first-year engineering students, but this pattern was evident in only one out of six spatial tasks administered. This highlights the variability of gender effects across different types of spatial measures. Similarly, Turgut and Yılmaz (2012) found no significant gender difference among pre-service primary mathematics teachers, while Korkmaz and Tekin (2020) reported comparable findings with prospective preschool teachers. These inconsistencies suggest that gender-related disparities in spatial ability may not be universal but rather influenced by contextual and demographic factors. Moreover, studies that approach spatial intelligence through the lens of multiple intelligences also report null findings. Gani et al. (2024), for instance, observed no gender difference in visual–spatial intelligence among elementary students, indicating that age and educational context may moderate such effects. Saad et al. (2024) further support this view by showing that gender differences tend to emerge more prominently in domains like communication and critical thinking than in spatial reasoning. In addition, the observed gender difference in this study may be partly explained by context-specific factors related to students’ everyday experiences and learning environments. In the Turkish context, differential engagement with spatially demanding activities such as digital games, navigation, or technology use may contribute to variations in spatial skill development. Furthermore, classroom practices and participation patterns in mathematics-related tasks may unintentionally reinforce these differences over time. Therefore, the observed disparity should be interpreted as a context-dependent outcome shaped by experiential and educational factors rather than as a fixed cognitive difference.

The Kruskal–Wallis *H* test indicated significant differences in spatial concept association scores across daily transportation modes [*H*(2) = 367.516, *p* < 0.001], with a large effect size (*ε*^2^ = 0.883). Students commuting by school bus outperformed those who walked or used private vehicles, and those using private vehicles scored higher than walkers. These results align with prior findings suggesting that transit-based travel, often involving navigation, timing, and decision-making, can enhance spatial cognition (Voss et al., 2015; Ermagun and Levinson, 2017). Although walking is generally associated with spatial development (Fang and Lin, 2017), its benefits may be limited in constrained or familiar environments, whereas transit use requires attention to spatial landmarks and route sequences. Furthermore, contextual factors such as built environment, travel distance, and household vehicle access may indirectly influence both transportation choices and spatial skill development (Zannat et al., 2020; Cunha and Cadima, 2024; Liu et al., 2024). These findings suggest that school transit, beyond its functional role, may serve as an important context for supporting students’ spatial concept associations.

The statistically significant difference observed in spatial concept association scores across disciplinary orientations, with mathematics-oriented participants outperforming those who identified with both mathematics and geography, reflects the differentiated spatial demands of academic domains. In mathematics education, spatial thinking supports understanding of graphs, geometric reasoning, and abstract visual models. Numerous studies confirm that mathematics students often develop strong spatial reasoning skills due to the visual and symbolic nature of their learning environments (Ishikawa and Newcombe, 2021; Del Cerro Velázquez and Méndez, 2021; Majeed and ALRikabi, 2022).

However, participants who reported dual orientation toward both mathematics and geography scored significantly lower than those aligned solely with mathematics. This finding may seem counterintuitive, but research suggests that different disciplines emphasize different types of spatial skills. While geography requires spatial interpretation in map reading and landform visualization, mathematics relies more on abstract spatial reasoning and visual problem-solving (Atit et al., 2020; McNeal and Petcovic, 2020). Thus, participants identifying with both domains might be engaging with a broader, yet potentially less specialized, spatial skill set.

Moreover, spatial skills are not equally cultivated across disciplines, even within STEM. Engineering and architecture programs often embed systematic training in spatial visualization, whereas geography and mathematics may approach spatial cognition through domain-specific content without generalized skill development (Porat and Ceobanu, 2024; Karpyuk and Davydenko, 2022). These differences in disciplinary emphasis might explain the small effect size observed and underscore the importance of targeted instructional strategies that explicitly support spatial reasoning aligned with disciplinary contexts.

Mathematics-oriented individuals performed better than dual-discipline participants. This illustrates how disciplinary identity can shape spatial concept associations. This supports the growing body of research advocating for discipline-specific spatial training to foster cognitive strengths and enhance academic outcomes in spatially intensive fields (Tomai et al., 2023; Lakin and Wai, 2020).

### 4.1 Limitations of the study

An important limitation of the present study is that the data collection process was limited to a single cross-sectional implementation. Although this design provided valuable insights into students’ disciplinary associations with spatial concepts, it did not allow for observing changes in these associations over time. In addition, the use of convenience sampling may limit the generalizability of the findings beyond the study context. Furthermore, while the associative-response method offers valuable insights into students’ immediate cognitive representations, it may not fully capture deeper or more stable conceptual understandings of spatial knowledge. Another limitation of the study is that group differences at the participant level were examined primarily using non-parametric comparisons. Although a multivariate model was evaluated to assess the simultaneous effects of potential confounding variables, the assumptions required for linear regression were not sufficiently met due to the bounded and derived nature of the dependent variable. Therefore, findings related to variables such as gender, mode of transportation, and disciplinary orientation should be interpreted as bivariate patterns rather than independent effects. Future research may benefit from employing more flexible modeling approaches that are better suited to handling bounded or non-normally distributed outcome variables.

### 4.2 Practical implications and recommendations for future research

The findings of this study have important implications for curriculum design and instructional practices in both mathematics and geography education. The observed differences in students’ disciplinary associations with spatial concepts indicate a need for more explicit integration of spatial reasoning across subject areas. Teachers can strengthen students’ conceptual understanding by incorporating interdisciplinary tasks that connect mathematical abstraction with geographic application, such as map-based problem-solving, spatial modeling, and coordinate-based analysis. In teacher education programs, structured opportunities for developing spatial thinking through digital mapping tools, GIS applications, and visual reasoning exercises may further enhance disciplinary transfer. In addition, considering the observed gender-based differences, educational practices should aim to provide more inclusive and equitable learning environments that support the development of spatial skills across all student groups.

For future research, longitudinal studies are recommended to examine how students’ spatial concept associations evolve through sustained instructional exposure. Comparative or cross-cultural investigations could also clarify how curricular frameworks shape disciplinary anchoring of spatial knowledge. In addition, mixed-method designs combining associative-response data with interviews, spatial performance tests, or eye-tracking measures would deepen understanding of how learners construct and apply spatial concepts across educational contexts.

## 5 Conclusion

This study makes three main contributions to the literature on spatial cognition and interdisciplinary learning. First, it introduces an associative-response approach as a methodological alternative to traditional performance-based spatial assessments, enabling the examination of how learners conceptually organize spatial knowledge. Second, it provides empirical evidence demonstrating that students’ spatial concept associations follow systematic disciplinary patterns rather than random distributions. Third, the findings extend theoretical discussions by suggesting that spatial cognition is not solely a domain-general ability but is partially shaped by discipline-oriented cognitive structures influenced by educational experiences.

Taken together, these findings highlight the importance of considering disciplinary context in the development and assessment of spatial thinking. Rather than treating spatial cognition as a uniform cognitive ability, the results suggest that learners’ conceptualizations are shaped by how spatial knowledge is structured and taught within different subject areas. This perspective has important implications for curriculum design, particularly in fostering interdisciplinary connections between mathematics and geography to support more integrated spatial understanding.

## Data Availability

The raw data supporting the conclusions of this article will be made available by the corresponding author upon reasonable request.
